# Interventions to improve comfort and physiological parameters in premature infants: a meta-analysis

**DOI:** 10.1590/1806-9282.20250493

**Published:** 2025-10-27

**Authors:** Doğan Çağrı Tanrıverdi, Fatma Şule Bilgiç, Aysu Yıldız Karaahmet

**Affiliations:** 1 Mehmet Akif Ersoy State Hospital, Department of Pediatric Cardiology – Çanakkale, Turkey.; 2 Çanakkale Onsekiz Mart University, Faculty of Health Sciences, Department of Midwifery – Çanakkale, Turkey.; 3 Biruni University, Faculty of Health Sciences, Department of Midwifery – Istanbul, Turkey.

## INTRODUCTION

Premature birth occurs due to various unpredictable factors, and infants born before 37 weeks of gestation are classified as "preterm."^1,2^ Each year, approximately 15 million preterm infants are born globally, with 65–75% of these births occurring late preterm in developed countries. Although data from developing nations is harder to obtain, it is assumed that the rates are similar to those in developed countries^1–3^. Preterm birth remains one of the primary causes of infant mortality and morbidity^3^.

Preterm infants in the neonatal intensive care unit (NICU) are exposed to stimuli such as bright lights, loud noises, and painful medical procedures, which can significantly impact their cognitive and behavioral development. This can result in adverse physiological changes such as heart rate (HR) fluctuations, oxygen saturation (SpO_2_) changes, and increased cortisol levels, as well as behavioral disruptions such as sleep–wake cycle disturbances, altered facial expressions, and crying patterns. These disruptions may negatively affect the neurodevelopment and overall stability of preterm infants, who are already prone to neurological and developmental issues^4,5^.

Assessing the physiological status of preterm infants is vital for understanding and supporting their regulation skills. Care models promoting neurological development are recommended to prevent early behavioral or developmental disorders^6^. Appropriately addressing behavioral conditions can positively influence neurodevelopment^6,7^. Meta-analysis studies on improving physiological parameters and comfort in preterm neonates in the NICU are limited. Recent systematic reviews and meta-analyses have examined the effectiveness of interventions such as maternal voice^8^, maternal care^9^, kangaroo care (KC)^10^, and music therapy^11^ on preterm infants. Stabilizing physiological parameters through non-invasive interventions can have long-term benefits for preterm neonates^12^. However, there is a lack of meta-analysis studies specifically focusing on non-pharmacological interventions in preterm infants. The aim of this study was to systematically review and meta-analyze the effects of interventions added to routine care for preterm infants, focusing on physiological parameters and comfort outcomes.

## METHODS

The preparation of the systematic review and meta-analysis adhered to the PRISMA guidelines^13^.

### Search strategy and selection criteria

The references for this systematic review were obtained through a search of electronic databases (PubMed/MEDLINE, Embase, PsycINFO, and Web of Science) conducted between September and October 2024, by reviewing the reference lists of the selected studies, and through consultations with experts in the field. PubMed/MEDLINE search terms were: "preterm" OR "prematurity" OR "baby" OR "newborn" OR "infant" AND "heart rate" AND "respiratory rate" AND "physiologic parameters" OR "oxygen saturation" OR "neonatal intensive care unit" AND "non-pharmacologic" [MeSH] OR "massage: [MeSH], OR "kangaroo care" [MeSH], while other databases were searched using equivalent search terms.

The following criteria, Participant, Intervention, Comparison, Outcomes and Study (PICOS) design, were taken into consideration in the selection of the studies to be included in the study:

Participant (P): The preterm infants included in the study met the following criteria for inclusion: (1) born before 37 weeks of gestation, (2) without a history of cerebral hemorrhage, neurological and central nervous system diseases, and convulsions,(3) not intubated, and (4) without sedative and analgesic drugs.

Intervention (I): Non-pharmacologic methods added to routine care: (1) position, (2) nest, (3) KC, and (4) music and auditory interventions.

Comparison (C): (1) Routine care and (2) crossover study.

Outcomes (O): (1) SpO_2_, (2) HR, (3) respiratory rate (RR), (4) body temperature, and (5) comfort.

Study design (S): Randomized controlled trials (RCTs) were included.

Studies were excluded if they met any of the following criteria: (1) articles that did not present original data; (2) observational studies; (3) studies that did not report on the outcomes of the intervention; (4) studies involving respiratory support, term infants, or painful stimuli; and (5) studies from which data on the treatment of preterm neonates could not be extracted.

### Data analysis

The impact of non-pharmacological interventions was assessed using the standardized mean difference (SMD) with 95% confidence intervals (95%CIs), as the physiological parameters and comfort outcomes were consistently measured across the included studies. The mean difference and standard deviation for physiological and comfort outcomes in both the intervention and control groups, as reported in the original studies, were entered into the Review Manager software (RevMan, version 5.4).

## RESULTS

### Literature review

A total of 584 studies were retrieved in the initial search, of which 389 articles with full-text access were reviewed. The 10 included RCTs are summarized in Table 1.

**Table 1:** Characteristics of included studies (n=10).

References\country	Population	Protocol	Comparisons
Altay and Küçükoğlu^14^ Turkey	89 (EG*: 44, CG*: 45)	The babies in the EG were immediately given a facilitated pinch position under a radiant heater.	Control: routine maintenance
Liao et al.^15^ China	103 (EG*: 34, CG*: 35)	The babies were divided into three groups: mother's voice, white noise, and a control group.	Control: routine maintenance
Parsa et al.^16^ Iran	100 (EG*: 50, CG*: 50)	The intervention group was given KMC* for 1 h per day for 7 days.	Control: routine maintenance
Manzotti et al.^17^ Italy	100 (EG*: 50, CG*: 50)	OMT intervention was applied once to the randomized infants in two groups.	Control: static touch
Jamehdar et al.^18^ Iran	70 (EG*: 36, CG*: 34)	A group of babies received KMC once, provided directly by their own mothers	Control: surrogate group
Tedesco et al.^19^ Brasilia	34 (EG*: 17, CG*: 17)	Hydrokinesiotherapy was applied to the babies in the intervention group, with two sessions lasting 10 min each, every other day.	Control: routine maintenance
Zengin and Cinar^20^ Turkey	60 (EG*: 30, CG*: 30)	The mothers of the babies in the intervention group provided KC by wearing aprons designed for Sarbebe KC	Control: standard KC
Çakıcı and Mutlu^21^ Turkey	20 (Group I–IV: 5)	The baby in each group was intervened in a different order on the right and left sides and face-down and back positions.	Crossover
Miranda et al.^22^ Brazil	27 (EG*: 13, CG*: 13)	Infants in the intervention group underwent KMC* and were administered 8–12 times over 24 h.	Control: routine maintenance
Karadag et al.^23^ Turkey	52 (EG*: 25, CG*: 7)	The babies were placed for 15 min in the nest where the device that simulated the sound of their mother's heart was located.	Control: routine maintenance

* EG: experimental group; CG: control group; KMC: kangaroo mother care; OMT: osteopathic manipulative treatment; KC: kangaroo care.

This systematic review and meta-analysis examined 10 studies, encompassing a total of 660 preterm infants, to systematically evaluate the impact of interventions added to the standard care of preterm infants on physiological outcomes and to assess the available evidence^14–23^. All studies included in the meta-analysis were RCT studies. All infants included in the studies were non-intubated infants between 24 and 37 gestational weeks (GWs), and the interventions were administered at least once for a maximum of 7 days.

### Physiological parameters

In the studies included in the analysis, the authors evaluated the effect of intervention results on physiological parameters and reported results related to SpO_2_, HR, RR, and body temperature. Subgroup analyses of physiologic parameters before and after intervention were presented as forest plots.

**Figure 1:**
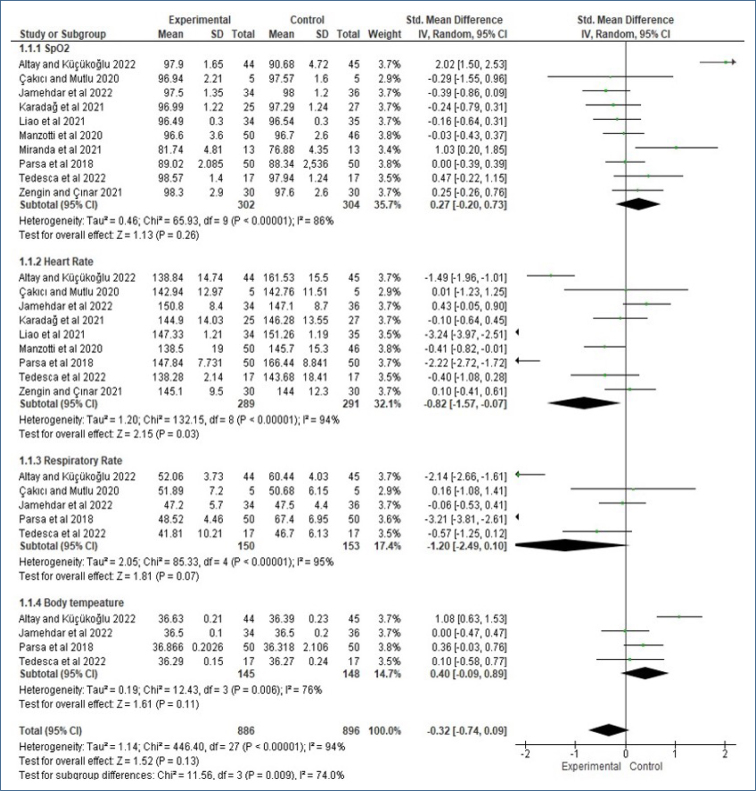
Subgroup meta-analysis results on the effect of non-pharmacological on the physiologic parameters.

According to the subgroup analysis of the combined results of the studies, there was no significant difference in physiological parameters in preterm infants (SMD: −0.32, 95%CI −0.74 to 0.09, Z=1.52, p=0.13, Figure 1). When analyzed in terms of effect size, it was observed that the effect size of HR and RR was negative, while it was positive for SpO_2_ and body temperature.

### Comfort

**Figure 2:**
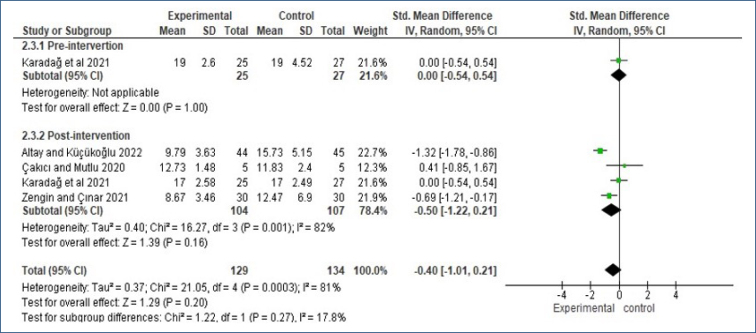
Subgroup meta-analysis results on the effect of non-pharmacological on the comfort.

Subgroup analyses of comfort values before and after intervention were presented as forest plots. When the post-intervention data were pooled, a high level of heterogeneity was found (I^2^=81%, p=0.0003). According to the subgroup analysis of the pooled results of the studies, there was no significant difference in comfort outcomes in preterm infants (SMD: −0.40, 95%CI −1.01 to 0.21, Z=1.29, p=0.20, Figure 2).

## DISCUSSION

The results of the study showed that interventions added to the care of preterm infants increased SpO_2_ and body temperature, while decreasing HR and RR, bringing them closer to normal values; however, did not affect the comfort of infants. This is important in terms of showing that non-invasive and effective behavioral interventions can be used to improve and maintain the health of infants.

The results of the analysis showed that there was no significant difference in physiological parameters between the interventions compared to the control group. However, in terms of effect size, SpO_2_ and body temperature increased, while HR and RR decreased. In a meta-analysis including 34 studies, it was reported that massage intervention applied to preterm infants positively affected the physiological parameters of preterm infants, and there was a significant difference compared to controls^10^. In another systematic review, it was reported that KC did not show significant changes in physiologic parameters^24^. In another systematic review analyzing 26 studies, it was reported that maternal voice stimulation increased SpO_2_ by decreasing HR^8^. Another meta-analysis analyzed 11 studies and reported that kangaroo care only increased SpO_2_ oxygen saturation in infants and had no effect on other physiological parameters^9^. In another meta-analysis study analyzing 14 RCTs and seven observational studies, it was reported that music therapy reduced RR and HR in newborns^11^. These findings differ because the interventions applied to preterm infants differ from each other. In addition, the difference in the prognosis of preterm infants should also be considered as a variable.

The results of the study showed that there was no significant difference between the groups in the comfort values of the interventions added to the routine care of preterm infants compared to the control group. In most of the studies included in the analysis, it was reported that interventions increased comfort compared to the control group. One study reported that the comfort levels were significantly higher in babies receiving KC combined with the wrapping method compared to the control group^21^. In another study, comfort levels were found to be higher when infants received blood group care compared to those positioned prone^25^. In another systematic review study, 26 research results were analyzed, and it was reported that maternal voice stimulation increased comfort, especially in painful procedures^8^. In the literature, meta-analytic studies examining the effect of interventions on comfort are very limited. The results of the existing analysis studies are contradictory. More studies are needed to increase the comfort levels of infants, which are related to physiologic and stress parameters.

Almost all of the studies included in this meta-analysis were assessed as having a high risk of bias due to lack of staff blinding. In contrast, one meta-analysis reported a low risk of bias among its included studies^8^. Another meta-analysis reported a high risk of bias, inconsistency, and uncertainty in included studies^11^. In another meta-analysis, the included studies were evaluated as having a high risk of bias^9^. Although study evaluations differ in the analyses, it can be said that the risk assessment of the studies included in this analysis is low.

The study's strengths include searching multiple databases, blinding researchers during data extraction, and ensuring low bias and error. Independent assessments and consensus-based decisions improved methodological quality. Additionally, the study provides up-to-date data on the physiological parameters and comfort outcomes of non-pharmacological interventions in preterm infants.

## CONCLUSION

The study found that interventions for preterm infants improved physiological parameters such as SpO_2_, body temperature, HR, and RR but did not affect their comfort. Most studies had a high risk related to lack of staff blinding but demonstrated a low risk of bias. Non-invasive interventions showed protective effects against neonatal adverse outcomes, with no harm. These results support the broader use of interventions such as KC in neonatal care.

## Data Availability

The datasets generated and/or analyzed during the current study are available from the corresponding author upon reasonable request.
